# Construction and validation of a machine learning–based prediction model for postoperative complications in patients with chronic otitis media

**DOI:** 10.3389/fmed.2026.1805224

**Published:** 2026-04-13

**Authors:** Jinlei Wang, Fuxun Zhang, Lijing Liu, Ye Ju, Chenguang Zhan

**Affiliations:** 1 Department of Eye, Ear, Nose, Throat and Oral Ward, No. 971 Hospital of People's Liberation Army Navy, Qingdao, China; 2 Department of Otolaryngology, No. 971 Hospital of People's Liberation Army Navy, Qingdao, China; 3 Department of Otolaryngology, Qingdao Municipal Hospital, Qingdao, China

**Keywords:** chronic otitis media, clinical multi-omics, machine learning, postoperative complications, prediction model, random forest

## Abstract

**Objective:**

A machine learning prediction model was developed and validated using Clinical Multi-omics (CMO) indicators to assess the risk of postoperative complications in patients with Chronic Otitis Media (COM). This model is intended to aid in perioperative management and individualized intervention.

**Methods:**

Patients were randomly allocated into a training set (*n* = 237) and a validation set (*n* = 101) in a 7:3 ratio. A total of 21 CMO indicators, including demographic, clinical, laboratory, and imaging data, were collected. In the training set, univariate analysis, Least Absolute Shrinkage and Selection Operator (LASSO) regression, and multivariate logistic regression were used to identify core predictive variables. Four machine learning models—Random Forest, Logistic Regression, K-Nearest Neighbors, and Gradient Boosting Machine—were constructed using these variables. Model performance was assessed using the area under the curve (AUC), calibration curves, and decision curve analysis (DCA). Interpretability was analyzed with SHapley Additive exPlanations (SHAP).

**Results:**

Multivariate analysis identified a history of diabetes, previous ear surgery, otorrhea, middle ear mucosal status, cholesteatoma presence, Eustachian tube function score, and preoperative C-reactive protein level as independent risk factors. Among the constructed models, the Random Forest model demonstrated superior overall performance, and the model was 0.885 in the training set and 0.853 in the validation set. The model also showed good calibration. DCA indicated significant clinical net benefit across a wide threshold probability range. SHAP analysis confirmed that a history of previous ear surgery and cholesteatoma presence were the most influential predictors.

**Conclusion:**

A machine learning-based prediction model for complications after COM surgery was developed and validated. The Random Forest model performed optimally, effectively predicting complication risk with favorable performance and considerable potential for clinical translation. It can serve as a promising tool for preoperative risk assessment and targeted postoperative monitoring.

## Introduction

Chronic Otitis Media (COM) is a common and potentially destructive chronic infection of the middle ear. It represents a leading cause of conductive hearing loss and imposes a significant global disease burden (1, 2). For patients with inadequate response to medical management, surgery is the primary treatment. However, the incidence of postoperative complications, such as infection, graft failure, and facial nerve injury, remains considerable. These complications significantly impact surgical outcomes and patient quality of life (3, 4). Currently, clinicians primarily rely on empirical experience to assess complication risks, lacking precise and quantitative prediction tools. This may lead to under-identification of high-risk patients or over-intervention in low-risk individuals (5).

The occurrence of postoperative complications is modulated by a complex interplay of multi-level factors. Patient comorbidities, such as a history of diabetes mellitus, are established risk factors due to their impact on healing and immune status (6). The severity of local disease is crucial. A history of previous ear surgery predicts more complex anatomy and a poorer healing foundation. The presence of otorrhea and the status of the middle ear mucosa directly reflect the activity of infection and inflammation. The presence of cholesteatoma significantly increases surgical difficulty and recurrence risk due to its invasive nature. Furthermore, Eustachian tube function is key to maintaining middle ear health; its dysfunction is an important mechanism in both the pathogenesis of otitis media and the development of postoperative complications. Systemic inflammatory markers, such as preoperative C-reactive protein (CRP), also indicate that the body’s overall inflammatory burden may affect postoperative recovery. However, the combined influence of these factors on complication risk is complex. Currently, an integrated prediction model incorporating demographic data, clinical characteristics, local pathological details, and laboratory indicators is lacking (7).

Machine learning algorithms can efficiently handle high-dimensional, non-linear clinical data and identify complex interactions among variables. They demonstrate significant advantages in constructing disease prediction models (8). Therefore, this study aimed to integrate multi-dimensional clinical, imaging, and laboratory data—termed Clinical Multi-omics (CMO). In the context of this study, CMO is operationally defined as the systematic integration of multi-phenotypic clinical data, with the core of capturing the complex interactions between different types of indicators. Unlike traditional clinical data integration that simply superimposes several single-dimensional indicators, this CMO framework emphasizes the holistic integration and synergistic analysis of multi-dimensional data, which can more comprehensively reflect the pathological characteristics of COM compared with the fragmented data integration in traditional research. Using advanced machine learning methods, we sought to develop and validate a comprehensive model for predicting the risk of postoperative complications in COM patients. This model is intended to provide a reliable basis for precise preoperative assessment and individualized perioperative management.

## Materials and methods

### Study population

We retrospectively enrolled patients with COM who underwent surgical treatment in the Department of Otorhinolaryngology-Head and Neck Surgery at our hospital between January 2021 and December 2023. The sample size calculation was based on an expected incidence of approximately 30–35% for the primary outcome (postoperative complications), adhering to the principle of “at least 10 events per variable” for prediction model development (4). Considering the number of core variables for multifactorial analysis and the requirements of machine learning modeling, and allowing for a 10% data missing rate, the minimum required sample size was determined to be 300. This study ultimately included 338 patients, which was deemed sufficient. The inclusion criteria were: (1) age ≥18 years; (2) confirmed diagnosis of COM (including cholesteatomatous and non-cholesteatomatous types) based on clinical and imaging findings and subsequent surgical treatment (9); (3) completion of all predefined baseline assessments preoperatively; (4) availability of complete follow-up data for complication adjudication. The exclusion criteria were: (1) concomitant active malignant tumors of the ear; (2) secondary middle ear pathologies due to non-inflammatory factors such as trauma or radiotherapy; (3) incomplete baseline assessments or follow-up data precluding outcome classification.

### Data collection

Multi-dimensional preoperative information was systematically collected from the hospital’s electronic medical record system, Picture Archiving and Communication System (PACS), and Laboratory Information System (LIS). Collected data included: (1) Demographic data: age and sex; (2) Comorbidity history: Diabetes Mellitus (DM); (3) Otologic history and characteristics: history of previous ear surgery, otorrhea status (dry/intermittent/continuous), middle ear mucosal status (normal/granulation/polypoid change), and presence or absence of cholesteatoma (10); (4) Functional and laboratory indicators: Eustachian tube function score and preoperative serum CRP level. All data were independently entered by two trained researchers, followed by consistency checks (11).

### Outcome definition

The primary outcome was the occurrence of any complication within 6 months postoperatively (12). Complications were defined as surgery-related adverse events, including but not limited to: postoperative infection (e.g., wound infection, acute otitis media), graft failure (e.g., tympanic membrane re-perforation), lack of hearing improvement or hearing deterioration, facial nerve injury, and vertigo (13). Outcome adjudication was independently performed by two attending physicians blinded to the patients’ baseline data, based on postoperative follow-up records. Cohen’s kappa coefficient was used to assess inter-rater reliability, with a value of 0.89 (*p* < 0.05) indicating almost perfect agreement between the two physicians. Any discrepancies were resolved by a third senior physician.

### Statistical analysis

Data analyses were performed using SPSS 26.0 and Python 3.8.5. Continuous data were presented as median (interquartile range) or mean ± standard deviation. Group comparisons were made using the Mann–Whitney U test or independent samples t-test. Categorical data were expressed as number (percentage) [*n* (%)], and group comparisons were conducted using the χ^2^ test or Fisher’s exact test. The overall cohort was randomly split into a training set and a validation set in a 7:3 ratio. In the training set, univariate analysis was first performed to screen for variables with *p* < 0.05. These variables were then subjected to variable selection via Least Absolute Shrinkage and Selection Operator (LASSO) regression. Subsequently, the selected variables were incorporated into a multivariable logistic regression to identify independent risk factors, and their odds ratios (OR) and 95% confidence intervals (CI) were calculated. Based on the core variables, four machine learning models—Random Forest, Logical Regression, K-Nearest Neighbors and Gradient Boosting Machine—were constructed using the scikit-learn library. Hyperparameters were optimized via 5-fold cross-validation (14). Model discrimination was evaluated using the receiver operating characteristic (ROC) curve and the area under the curve (AUC). Delong Test was used to compare the statistical significance of AUC differences between models. Model calibration was assessed using calibration curves. Clinical net benefit was analyzed via decision curve analysis (DCA). Finally, SHapley Additive exPlanations (SHAP) values were employed to interpret the prediction logic of the optimal model from both global and local perspectives.

## Results

### Comparison of baseline characteristics between the training and validation sets

No statistically significant differences (*p* > 0.05) were observed between the training and validation sets regarding demographic characteristics (age, sex, body mass index, smoking history, diabetes history, etc.), clinical disease features (disease duration, otorrhea status, type of hearing loss, hearing threshold levels, tympanic membrane and middle ear pathology, etc.), surgical information (surgical approach), imaging features (mastoid pneumatization type, ossicular erosion), or laboratory and microbiological indicators (preoperative secretion culture, inflammatory markers, etc.). This indicates a balanced and well-comparable dataset split (Table 1).

**Table 1:** Comparison of baseline characteristics between the training and validation sets.

Variables		Training set (*n* = 237)	Validation set (*n* = 101)	*t/χ^2^*	*p*
Age (years)		46.81 ± 15.21	47.51 ± 14.18	0.391	0.696
Sex (Male/Female)		121 (51.05%)/116 (48.95%)	55 (54.46%)/46 (45.54%)	0.328	0.567
Smoking history (Yes/No)		75 (31.65%)/162 (68.35%)	35 (34.65%)/66 (65.35%)	0291	0.589
Diabetes history (Yes/No)		31 (13.08%)/206 (86.92%)	11 (10.89%)/90 (89.11%)	0.312	0.578
Previous ear surgery (Yes/No)		83 (35.02%)/154 (64.98%)	38 (37.62%)/63 (62.38%)	0.209	0.648
Disease duration (years)		11.72 ± 9.31	12.43 ± 10.12	0.626	0.532
Body mass index (kg/m^2^)		23.51 ± 3.42	23.81 ± 3.61	0.265	0.792
Otorrhea status	Dry ear	142 (59.92%)	62 (61.39%)	0.103	0.950
	Intermittent otorrhea	65 (27.43%)	26 (25.74%)		
	Persistent otorrhea	30 (12.66%)	13 (12.87%)		
Hearing loss type (conductive/mixed)		152 (64.14%)/85 (35.86%)	68 (67.33%)/33 (32.67%)	0.318	0.573
Air conduction pure-tone average (dB HL)		51.62 ± 14.73	52.91 ± 15.32	0.728	0.467
Air-bone gap (dB)		31.81 ± 11.42	32.56 ± 10.95	0.559	0.576
Tympanic membrane perforation type	Central	124 (52.32%)	56 (55.45%)	0.507	0.776
	Marginal/Attic	87 (36.71%)	33 (32.67%)		
	Total perforation	26 (10.97%)	12 (11.88%)		
Middle ear mucosa status	Good/Mild Edema	92 (38.82%)	42 (41.58%)	0.281	0.869
	Granulation tissue	108 (45.57%)	43 (42.57%)		
	Polypoid change	37 (15.61%)	16 (15.84%)		
Cholesteatoma presence (Yes/No)		89 (37.55%)/148 (62.45%)	35 (34.65%)/66 (65.35%)	0.256	0.613
Eustachian tube function score		4.51 ± 1.81	4.31 ± 1.92	0.919	0.359
Surgical approach	Tympanoplasty alone	105 (44.30%)	49 (48.51%)	0.509	0.775
	Mastoidectomy + Tympanoplasty	97 (40.93%)	38 (37.62%)		
	Other/More complex procedures	35 (14.77%)	14 (13.86%)		
Mastoid pneumatization type (CT)	Pneumatic	108 (45.57%)	49 (48.51%)	0.484	0.785
	Diploic	94 (39.66%)	36 (35.64%)		
	Sclerotic	35 (14.77%)	16 (15.84%)		
Ossicular chain erosion (CT) (Yes/No)		131 (55.27%)/106 (44.73%)	58 (57.43%)/43 (42.57%)	0.133	0.715
Preoperative secretion culture	Negative	124 (52.32%)	56 (55.45%)	0.307	0.858
	Gram-positive bacteria	67 (28.27%)	26 (25.74%)		
	Gram-negative bacteria	46 (19.41%)	19 (18.81%)		
Preoperative white blood cell count (×10^9^/L)		7.05 ± 2.21	7.28 ± 2.41	0.853	0.394
Preoperative neutrophil percentage (%)		63.21 ± 11.52	64.52 ± 10.81	0.974	0.331
Preoperative CRP (mg/L)		9.62 ± 8.25	10.32 ± 8.94	0.696	0.487

CT, computed tomography; CRP, C-reactive protein.

### Univariate analysis of postoperative complications in patients with chronic otitis media based on the training set

Among the 237 patients in the training set who underwent surgery for COM, they were categorized into a complication group (*n* = 71) and a non-complication group (*n* = 166) based on the occurrence of postoperative complications. Univariate analysis revealed that seven factors were significantly associated with the incidence of postoperative complications (all *p* < 0.05): history of diabetes mellitus, previous ear surgery, otorrhea status (particularly persistent otorrhea), middle ear mucosal status (especially polypoid change), presence of cholesteatoma, Eustachian tube function score, and preoperative CRP. The remaining indicators showed no statistically significant differences between the two groups (all *p* > 0.05) (Table 2).

**Table 2:** Univariate analysis of factors influencing postoperative complications in chronic otitis media within the training set.

Variables		Complication group (*n* = 71)	Non-complication group (*n* = 166)	*t/χ^2^*	*p*
Age (years)		48.21 ± 14.71	46.21 ± 15.42	0.928	0.355
Sex (Male/Female)		38 (53.5%)/33 (46.5%)	83 (50.0%)/83 (50.0%)	0.247	0.619
Smoking history (Yes/No)		26 (36.6%)/45 (63.4%)	49 (29.5%)/117 (70.5%)	1.159	0.282
Diabetes history (Yes/No)		18 (25.4%)/53 (74.6%)	11 (6.6%)/155 (93.4%)	16.238	<0.001*
Previous ear surgery (Yes/No)		35 (49.3%)/36 (50.7%)	33 (19.9%)/133 (80.1%)	21.033	<0.001*
Disease duration (years)		12.52 ± 9.81	11.32 ± 9.12	0.907	0.365
Body mass index (kg/m^2^)		23.21 ± 3.61	23.62 ± 3.35	0.81.	0.401
Otorrhea status	Dry ear	28 (39.4%)	110 (66.3%)	38.308	<0.001*
	Intermittent otorrhea	20 (28.2%)	43 (25.9%)		
	Persistent otorrhea	23 (32.4%)	13 (7.8%)		
Hearing loss type (Conductive/Mixed)		48 (67.6%)/23 (32.4%)	104 (62.7%)/62 (37.3%)	0.531	0.466
Air conduction pure-tone average (dB HL)		53.81 ± 15.11	50.21 ± 14.42	1.735	0.084
Air-bone gap (dB)		32.81 ± 11.81	30.91 ± 11.11	1.183	0.238
Tympanic membrane perforation type	Central	32 (45.1%)	92 (55.4%)	2.136	0.344
	Marginal/Attic	30 (42.3%)	57 (34.3%)		
	Total perforation	9 (12.7%)	17 (10.2%)		
Middle ear mucosa status	Good/Mild Edema	20 (28.2%)	72 (43.4%)	25.713	<0.001***
	Granulation tissue	27 (38.0%)	77 (46.4%)		
	Polypoid change	24 (33.8%)	17 (10.2%)		
Cholesteatoma presence (Yes/No)		40 (56.3%)/31 (43.7%)	49 (29.5%)/117 (70.5%)	15.234	<0.001*
Eustachian tube function score		5.41 ± 1.69	4.12 ± 1.61	5.566	<0.001*
Surgical approach	Tympanoplasty alone	27 (38.0%)	78 (47.0%)	1.701	0.427
	Mastoidectomy + Tympanoplasty	33 (46.5%)	64 (38.6%)		
	Other/More complex procedures	11 (15.5%)	24 (14.5%)		
Mastoid pneumatization type (CT)	Pneumatic	27 (38.0%)	81 (48.8%)	2.535	0.282
	Diploic	31 (43.7%)	63 (38.0%)		
	Sclerotic	13 (18.3%)	22 (13.3%)		
Ossicular chain erosion (CT) (Yes/No)		45 (63.4%)/26 (36.6%)	86 (51.8%)/80 (48.2%)	2.694	0.101
Preoperative secretion culture	Negative	33 (46.5%)	91 (54.8%)	1.796	0.407
	Gram-positive bacteria	21 (29.6%)	46 (27.7%)		
	Gram-negative bacteria	17 (23.9%)	29 (17.5%)		
Preoperative white blood cell count (×10^9^/L)		7.41 ± 2.51	6.97 ± 2.01	1.429	0.154
Preoperative neutrophil percentage (%)		65.31 ± 10.82	62.78 ± 11.15	1.614	0.108
Preoperative CRP (mg/L)		13.81 ± 8.11	7.56 ± 6.91	6.048	<0.001*

CT, computed tomography; CRP, C-reactive protein. *p < 0.05.

### Multivariate logistic regression analysis for risk of postoperative complications based on the training set

**Figure 1:**
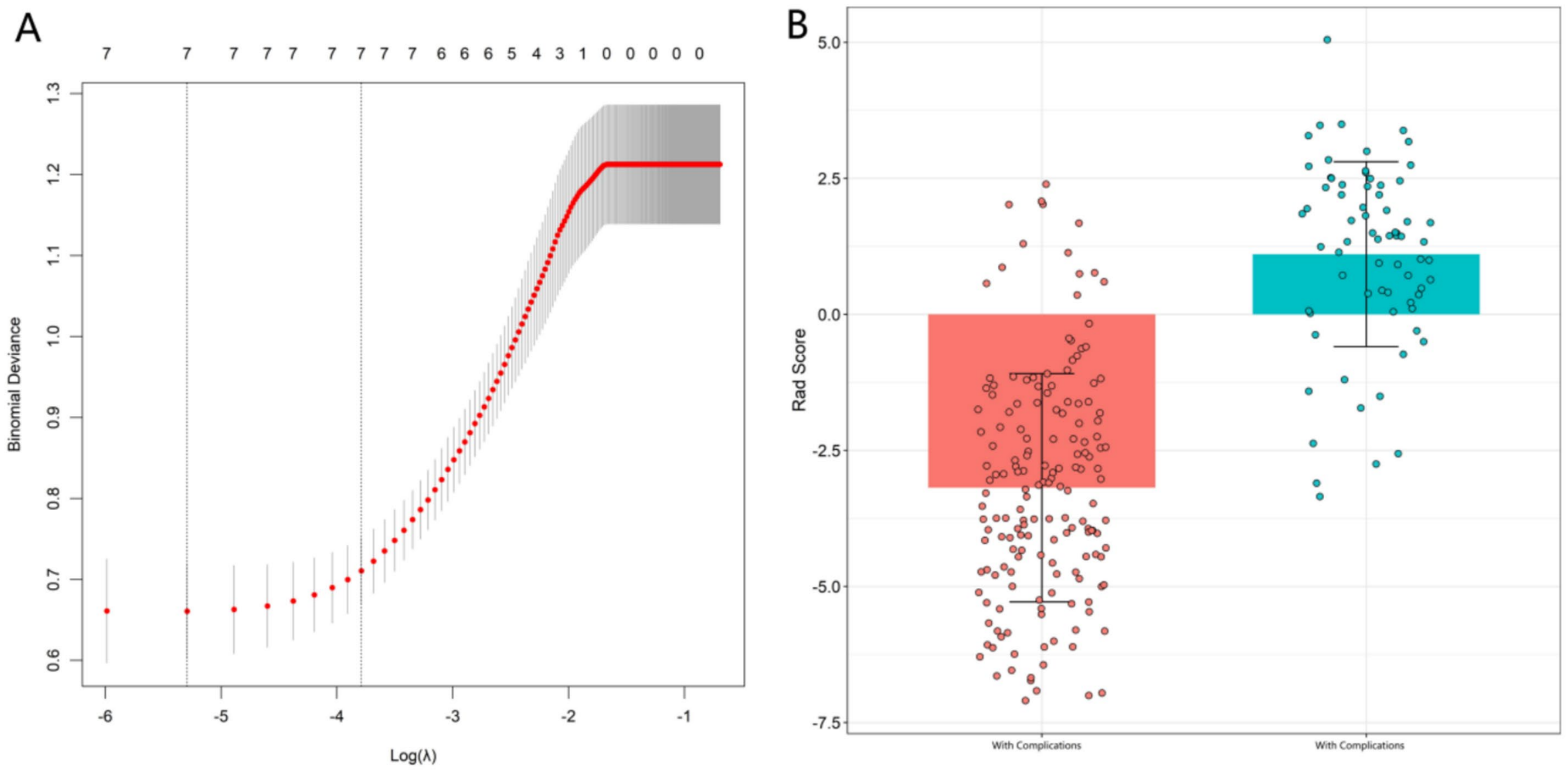
LASSO regression plot **(A)** and comparison of LASSO score differences **(B)**.

Using the occurrence of complications as the dependent variable (1 = complication group, 0 = non-complication group) (Supplementary Table 1), the seven statistically significant indicators from the univariate analysis were first subjected to variable selection via LASSO regression. The LASSO regression results (Figure 1) indicated that all seven predictors were retained (with non-zero coefficients) for the subsequent multivariate analysis.

The results of the multivariate logistic regression analysis demonstrated that history of diabetes mellitus, previous ear surgery, otorrhea status, middle ear mucosal status, presence of cholesteatoma, Eustachian tube function score, and preoperative CRP were all independent risk factors for postoperative complications (*p* < 0.05). Specifically, diabetes history (OR = 1.996), previous ear surgery (OR = 3.148), otorrhea status (OR = 1.494), middle ear mucosal status (OR = 1.839), presence of cholesteatoma (OR = 1.864), Eustachian tube function score (OR = 1.662), and preoperative CRP (OR = 1.143) increased the risk of complications (Table 3).

**Table 3:** Multivariate logistic regression analysis for the risk of postoperative complications based on the training set.

Factor	*β*	*SE*	*Wald*	*p*	*OR*	95%CI
Diabetes history	0.691	0.213	10.58	0.018*	1.996	1.315–3.029
Previous ear surgery	1.189	0.302	15.47	<0.001*	3.148	1.711–3.452
Otorrhea status	0.402	0.189	4.56	0.012*	1.494	1.041–1.487
Middle ear mucosal status	0.609	0.219	7.731	0.047*	1.839	1.173–1.829
Presence of cholesteatoma	0.623	0.205	9.94	0.045*	1.864	1.129–1.857
Eustachian tube function score	0.508	0.139	13.433	0.002*	1.662	1.267 ~ 2.180
Preoperative CRP	0.134	0.032	17.697	<0.001*	1.143	1.074 ~ 1.217

CRP, C-reactive protein; β, regression coefficient; SE, standard error; OR, odds ratio; CI, confidence interval. *p < 0.05.

### Machine learning model performance evaluation

**Figure 2:**
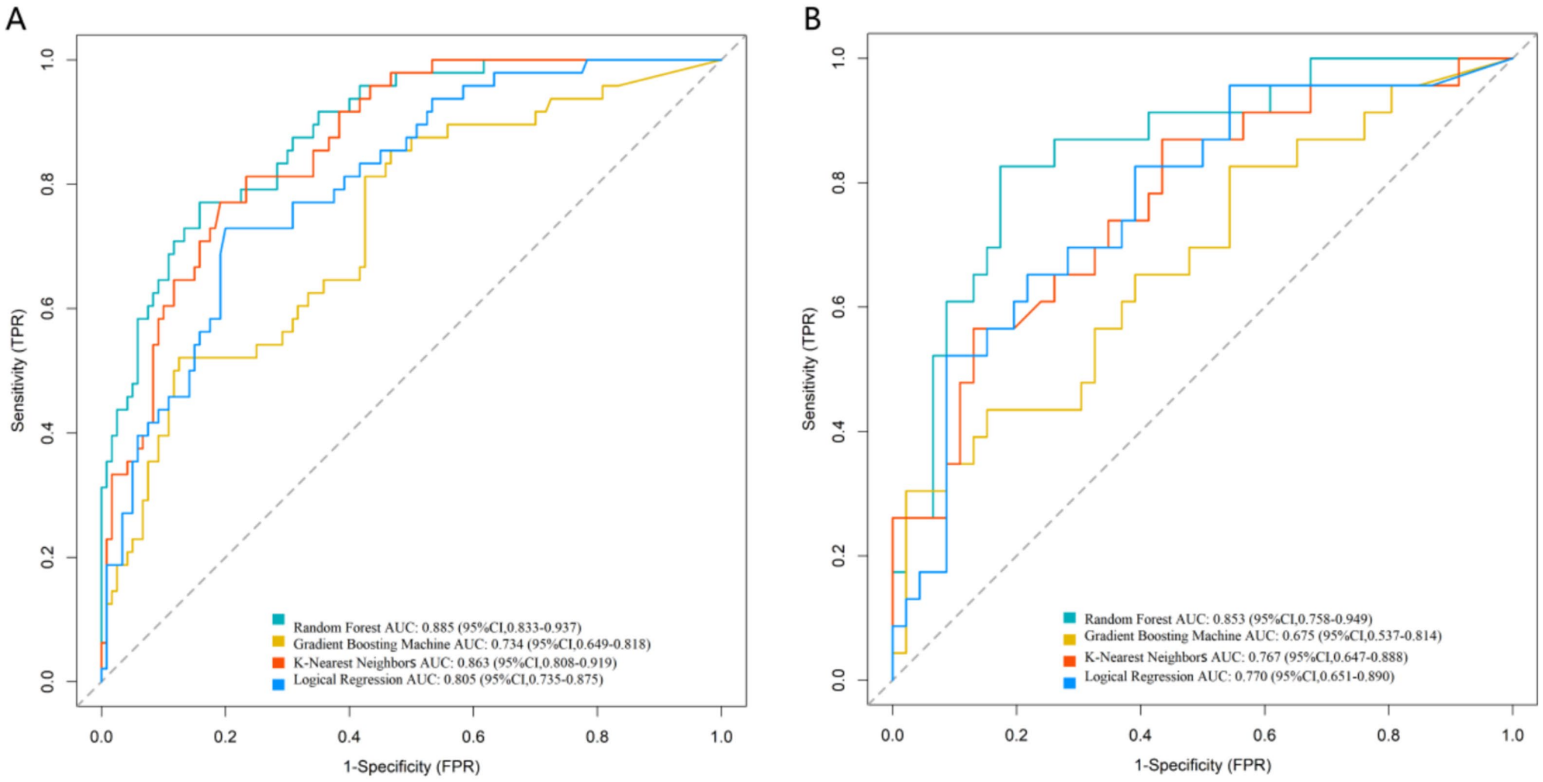
Receiver operating characteristic curve analysis of the prediction model in the training **(A)** and validation **(B)** sets.

ROC curve analysis (Figure 2) showed that in the training set, the Random Forest model demonstrated the best discriminatory ability, with an AUC of 0.885 (95% CI: 0.833–0.937). The AUC values for the other models were as follows: Logical Regression (0.805, 95% CI: 0.735–0.875), Gradient Boosting Machine (0.734, 95% CI: 0.649–0.818), and K-Nearest Neighbors (0.863, 95% CI: 0.808–0.919). In the independent validation set, the Random Forest achieved the highest AUC (0.853, 95% CI: 0.758–0.949), while the Gradient Boosting Machine (0.675, 95% CI: 0.537–0.814), Logical Regression (0.770, 95% CI: 0.651–0.890), and K-Nearest Neighbors (0.767, 95% CI: 0.647–0.888) performed similarly. The results of DeLong test showed no significant difference in AUC among the four models (all *p* > 0.05). This indicates that all models possessed good and stable discriminatory capability for postoperative complication risk.

**Figure 3:**
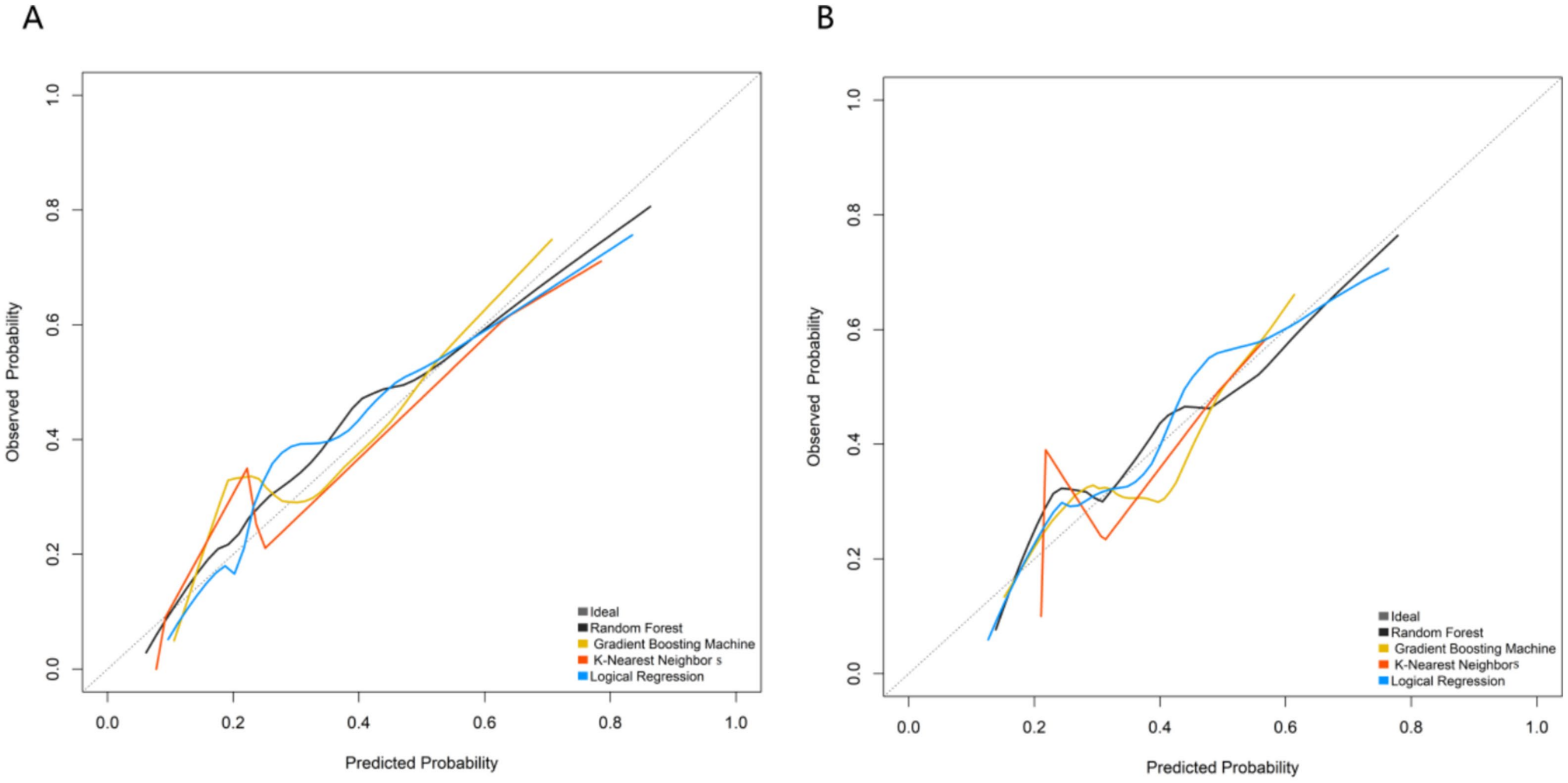
Calibration curves of predictive models in training set **(A)** and validation set **(B)**.

Calibration curves assessed the agreement between predicted probabilities and observed outcomes (Figure 3). In both the training and validation sets, the calibration curves for all four models closely approximated the ideal diagonal line. Quantitative evaluation of calibration was performed using the Brier score (for all machine learning models): the Random Forest model achieved the lowest Brier scores (0.082 in the training set, 0.095 in the validation set), with values far below 0.2 indicating excellent calibration; all other models had Brier scores ranging from 0.08 to 0.11. Among them, the Random Forest model’s calibration curve was closest to the diagonal in both datasets, demonstrating the best predictive calibration, meaning its predicted risk probabilities highly aligned with the actual risk.

**Figure 4:**
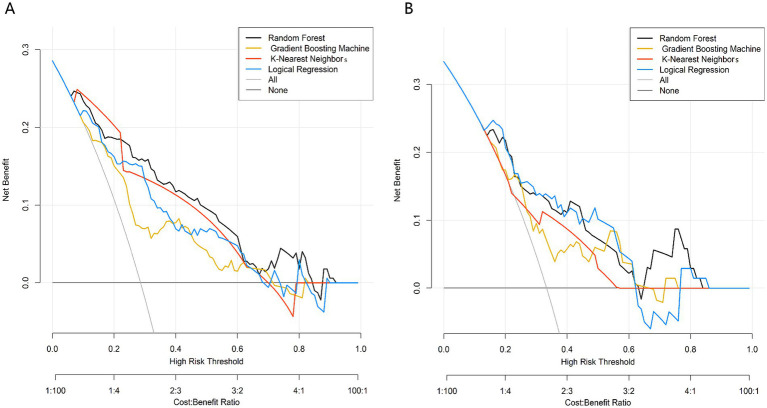
Decision curve of predictive models in training set **(A)** and validation set **(B)**.

DCA was used to evaluate the clinical net benefit of the models across different risk thresholds (Figure 4). The analysis revealed that across a wide range of clinical decision thresholds in both datasets, the net benefit of using any of the four machine learning models for decision-making was significantly higher than the extreme strategies of “intervene on all patients” or “intervene on no patients.” This confirms the practical clinical application value of the developed models. In both datasets, the RF model maintained a stable and high net benefit level within the core clinical decision interval, further supporting its clinical utility.

In summary, based on a comprehensive evaluation of AUC, calibration, and clinical net benefit, the Random Forest model demonstrated robust and excellent overall performance in predicting the risk of postoperative complications in COM patients. It was therefore selected as the base algorithm for the final integrated CMO prediction model.

### Evaluation of model predictability interpretability

To enhance the clinical interpretability of the optimal model, this study employed a Nomogram to visualize the prediction logic of the RF model. Additionally, SHAP values were used to quantitatively assess the global contribution of each feature, thereby elucidating the model’s decision-making basis.

**Figure 5:**
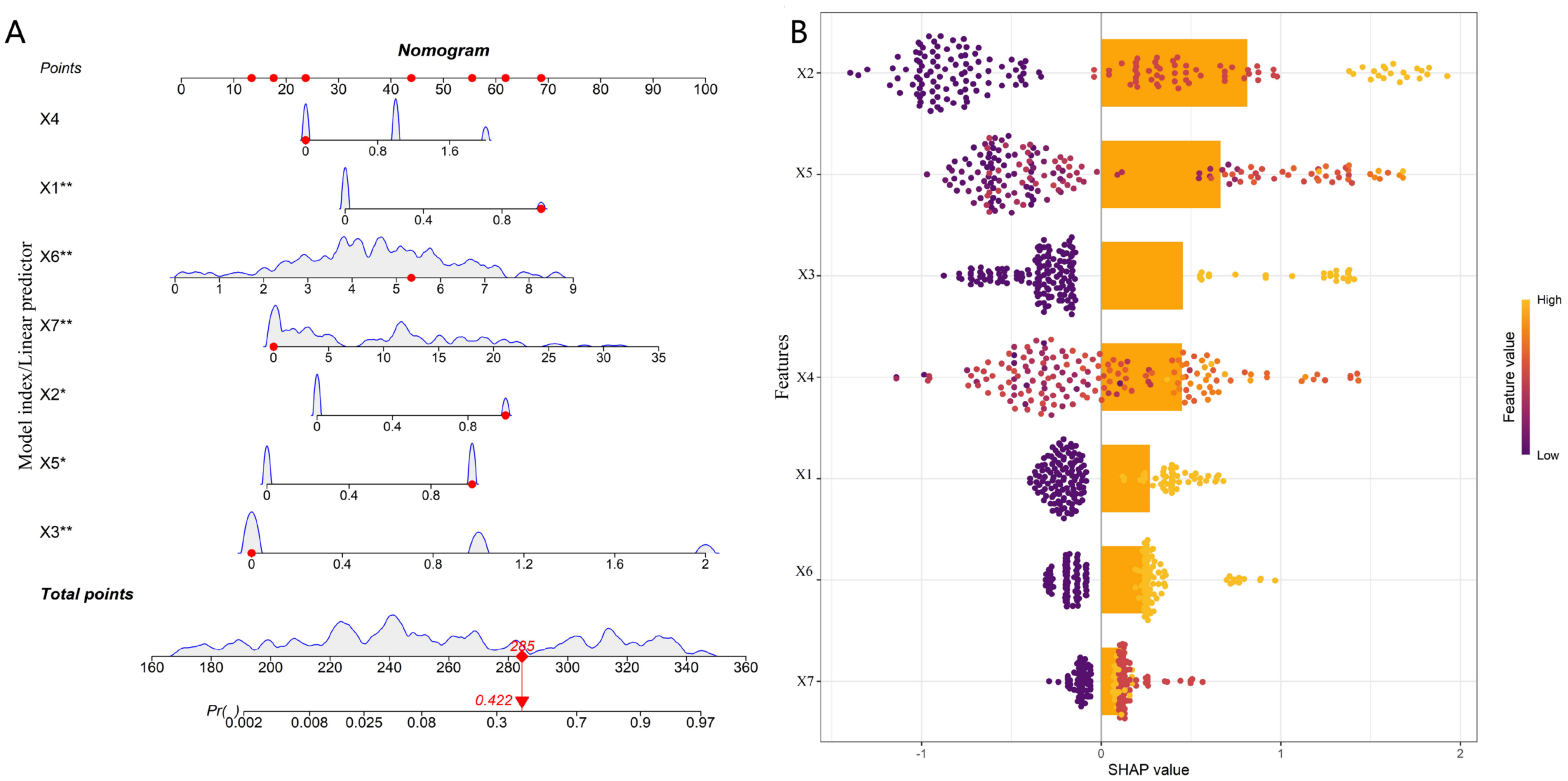
Model interpretability analysis: **(A)** Nomogram for predicting postoperative complication risk and **(B)** global feature importance ranking based on SHAP values. X1, diabetes history; X2, previous ear surgery; X3, otorrhea status; X4, middle ear mucosal status; X5, presence of cholesteatoma; X6, eustachian tube function score; X7, preoperative C-reactive protein.

As shown in Figure 5A, the constructed Nomogram integrates the seven core predictor variables, providing clinicians with an intuitive tool for individualized prediction. All variables in the model were identified as risk factors, where an increase in their value elevates the predicted probability of postoperative complications. Specifically, previous ear surgery (X2) and the presence of cholesteatoma (X5) were assigned higher weights in the Nomogram, indicating their greater contribution to risk prediction. Diabetes history (X1), otorrhea status (X3), and middle ear mucosal status (X4) were of moderate importance. Eustachian tube function score (X6) and preoperative CRP (X7), as continuous variables, contributed to a cumulative risk score as their values increased. Users can locate a patient’s values for each feature on the Nomogram, sum the corresponding points, and read the individualized predicted probability of complications on the total points and probability (Pt) axis at the bottom.

To further quantify the relative importance of each feature to the model’s predictions, a SHAP analysis was performed (Figure 5B). The global feature importance ranking revealed the following order of contribution to the model’s output: previous ear surgery (X2) > presence of cholesteatoma (X5) > otorrhea status (X3) > middle ear mucosal status (X4) > diabetes history (X1) > Eustachian tube function score (X6) > preoperative CRP (X7). This ranking was highly consistent with the trend of effect sizes (ORs) in the multivariate logistic regression analysis and the weight assignment of variables in the Nomogram. Together, they reveal that previous ear surgery and the presence of cholesteatoma are the most critical clinical risk factors driving the model’s predictions, with an influence surpassing other inflammatory markers and functional scores.

In conclusion, by combining the Nomogram and SHAP analysis, this section not only provides a user-friendly visual risk prediction tool but also quantitatively parses and confirms the hierarchy and direction of contributions of clinical and laboratory indicators within the prediction model from a machine learning perspective. This dual approach of “visual tool” and “intrinsic mechanism explanation” significantly enhances the transparency and clinical acceptability of the complex prediction model, laying a solid foundation for its translational application in postoperative risk management for COM patients.

## Discussion

This study successfully developed and validated a risk prediction model for postoperative complications by integrating multidimensional clinical indicators, based on a cohort of 338 patients undergoing surgery for COM. Among the models evaluated, the Random Forest model demonstrated the most optimal performance, achieving AUC of 0.885 and 0.853 for predicting complications in the training and validation sets, respectively. Furthermore, calibration curves and DCA confirmed the model’s good calibration and clinical utility. By employing the SHAP framework to interpret the model’s decision logic, we identified seven core predictive factors: history of diabetes, previous ear surgery, otorrhea status, middle ear mucosal status, presence of cholesteatoma, Eustachian tube function score, and preoperative CRP level. This provides a crucial quantitative tool for preoperative risk assessment in clinical practice.

The pathophysiological mechanisms of the seven identified core predictors are closely associated with surgical trauma, tissue healing, infection control, and the local microenvironment (15). Previous ear surgery was identified as the most influential risk factor with the highest SHAP value, and its dominant impact is attributed to the irreversible structural and microenvironmental damage caused by prior otologic surgery. Prior surgical interventions lead to disruption of the anatomies in the middle ear and mastoid region, scar adhesion, and compromised blood supply. These alterations not only significantly increase the difficulty and trauma of revision surgery but also severely impair the local microenvironment for tissue healing (16). Presence of cholesteatoma, another high-risk factor, exhibits invasive growth characteristics that often result in extensive bone destruction and are frequently associated with refractory infections (17). otorrhea status and middle ear mucosal status serve as direct indicators of the activity and severity of middle ear infection (18). History of diabetes, as a systemic metabolic factor, significantly increases the risk of postoperative infection and poor healing by impairing microcirculation, weakening tissue repair capacity, and reducing immune defense function (19). An elevated Eustachian tube function score directly indicates dysfunction in middle ear ventilation and drainage (20). An elevated preoperative CRP level, a sensitive marker of systemic inflammatory response, suggests that the patient is in a state of systemic inflammation (21).

Regarding model performance, the Random Forest model proved to be the most robust among the four machine learning algorithms tested. Its AUC remained at 0.853in the independent validation set, and its calibration curve was the closest to the ideal diagonal in both the training and validation sets. DCA further confirmed that across a wide range of clinical decision thresholds, the use of this model for risk stratification provided a greater clinical net benefit compared to the simple strategies of either intensifying monitoring for all patients or intervening on none. The application of SHAP analysis, particularly its revelation of the feature importance ranking “previous far Surgery>presence of cholesteatoma,” enhanced the transparency of the model’s decision-making logic.

The innovations of this study are threefold. First, it is the first to systematically construct and compare multiple machine learning prediction models for this purpose. Second, the modeling process was rigorous, adhering to a complete pathway of “univariate screening → confirmation by multivariate logistic regression → machine learning modeling and validation → interpretability analysis.” Third, by combining Nomogram and SHAP analysis, the study achieved a unification of tool practicality and theoretical interpretability.

This study also has several limitations. First, as a single-center retrospective study, the model constructed in this research has only undergone internal validation with a 7:3 training/validation set split and no external validation has been performed yet, which leads to relatively limited external validity and generalizability of the findings; the external validity of the model thus needs to be further verified and calibrated through well-designed multi-center, prospective cohort studies in the future. Second, all predictor variables were preoperative baseline indicators; future research could explore the development of dynamic prediction models that incorporate intraoperative and postoperative information. Third, the prediction endpoint of the model was a composite event of “complication occurrence”; future studies could further investigate its predictive capability for specific types of complications. In addition, a large number of hierarchical data with certain clinical subjectivity were included in the predictive factors of the model; in the subsequent construction and optimization, we will incorporate an appropriate amount of count data and objective evaluation indicators (such as objective detection values of Eustachian tube function and quantitative CT scores of the middle ear) to improve the standardization and clinical promotion value of the model.

In conclusion, the integrated prediction model based on the Random Forest algorithm developed in this study demonstrates good predictive performance for the risk of postoperative complications in COM patients. This model provides clinicians with a potential quantitative tool to support personalized preoperative risk assessment.

## Data Availability

The raw data supporting the conclusions of this article will be made available by the authors, without undue reservation.
