# Supplementary material for: Construction and validation of a machine learning–based prediction model for postoperative complications in patients with chronic otitis media
Source: Front Med (Lausanne). 2026 Apr 13;13:1805224. doi: 10.3389/fmed.2026.1805224 (PMC13110985; doi:10.3389/fmed.2026.1805224)
Supplement: Supplementary file 1 [file Table_1.DOCX]

**Supplemental Table 1.** Variable assignment table

| Variable | Meaning | Assignment |
| --- | --- | --- |
| X1 | Diabetes History | 1=Yes, 0=No |
| X2 | Previous Ear Surgery | 1=Yes, 0=No |
| X3 | Otorrhea Status | 0=Dry ear, 1=Intermittent otorrhea, 2=Persistent otorrhea |
| X4 | Middle Ear Mucosal Status | 0=Good/Mild edema, 1=Granulation tissue, 2=Polypoid change |
| X5 | Presence of Cholesteatoma | 1=Yes, 0=No |
| X6 | Eustachian Tube Function Score | Continuous variable |
| X7 | Preoperative CRP | Continuous variable |
| Y | Treatment Outcome | 1=Complication group, 0=Non-complication group |
